# Nest Suitability, Fine-Scale Population Structure and Male-Mediated Dispersal of a Solitary Ground Nesting Bee in an Urban Landscape

**DOI:** 10.1371/journal.pone.0125719

**Published:** 2015-05-07

**Authors:** Margarita M. López-Uribe, Stephen J. Morreale, Christine K. Santiago, Bryan N. Danforth

**Affiliations:** 1 Department of Entomology, Cornell University, Ithaca, New York, 14853, United States of America; 2 Department of Natural Resources, Cornell University, Ithaca, New York, 14853, United States of America; Australian National University, AUSTRALIA

## Abstract

Bees are the primary pollinators of flowering plants in almost all ecosystems. Worldwide declines in bee populations have raised awareness about the importance of their ecological role in maintaining ecosystem functioning. The naturally strong philopatric behavior that some bee species show can be detrimental to population viability through increased probability of inbreeding. Furthermore, bee populations found in human-altered landscapes, such as urban areas, can experience lower levels of gene flow and effective population sizes, increasing potential for inbreeding depression in wild bee populations. In this study, we investigated the fine-scale population structure of the solitary bee *Colletes inaequalis* in an urbanized landscape. First, we developed a predictive spatial model to detect suitable nesting habitat for this ground nesting bee and to inform our field search for nests. We genotyped 18 microsatellites in 548 female individuals collected from nest aggregations throughout the study area. Genetic relatedness estimates revealed that genetic similarity among individuals was slightly greater within nest aggregations than among randomly chosen individuals. However, genetic structure among nest aggregations was low (Nei’s G*_ST_* = 0.011). Reconstruction of parental genotypes revealed greater genetic relatedness among females than among males within nest aggregations, suggesting male-mediated dispersal as a potentially important mechanism of population connectivity and inbreeding avoidance. Size of nesting patch was positively correlated with effective population size, but not with other estimators of genetic diversity. We detected a positive trend between geographic distance and genetic differentiation between nest aggregations. Our landscape genetic models suggest that increased urbanization is likely associated with higher levels of inbreeding. Overall, these findings emphasize the importance of density and distribution of suitable nesting patches for enhancing bee population abundance and connectivity in human dominated habitats and highlights the critical contribution of landscape genetic studies for enhanced conservation and management of native pollinators.

## Introduction

Bees are the main pollinators of flowering plants in natural and agricultural areas [1,2]. About 35% of the world’s most important crops rely on bee pollination for successful reproduction and 70% of crops exhibit increased fruit size and yield when visited by bees [3–5]. Worldwide trends of bee declines are raising concerns about the economic and environmental consequences associated with the loss of the pollination services they provide [6,7]. Multiple studies over the past 10 years have identified three major drivers of decline in bee population abundance: (1) environmental stressors, including habitat loss and pesticide exposure; (2) pathogens and parasites; and (3) loss of genetic diversity [8]. While the effects of habitat loss, pesticide exposure and pathogen loads are moderately understood in wild bees [9–11], the levels and patterns of genetic diversity [12], and the processes that determine these patterns (e.g. gene flow) remain relatively unexplored in wild bees [13].

For many bee species, genetic population structure at fine geographic scales is unexpected due to their high mobility and potential for long distance dispersal. Even though species in highly fragmented habitats can show moderate to high levels of genetic differentiation [14,15], studies of population structure in bees generally indicate low genetic differentiation at regional and even continental geographic scales [16–20]. By contrast, other bee species exhibit strong philopatry and intranidal mating behavior that can lead to high levels of inbreeding [21,22]. Thus, the behavioral ecology of these bee species could generate population structure at fine geographic scales despite signals of gene flow at larger geographic scales.

Fine-scale population structure as a result of philopatric behavior can provide evolutionary benefits under certain conditions. For example, to maintain local adaptation to environmental conditions [23], decrease high costs of dispersal [24], increase probability of mate encounter when population densities are low [25], and enhance cooperation among members of a social group [26]. However, fine-scale structures increase the risk of extreme coancestry within populations and can lead to inbreeding depression, a reduction in individual fitness due to increasing levels of genetic homozygosity and expression of partially deleterious alleles [27]. The negative effects of inbreeding depression on fitness have been demonstrated in many species of mammals [28], birds [29], plants [30] and in threatened populations with low effective population sizes [31].

Many characteristics of bees make them particularly prone to inbreeding depression under conditions of low genetic diversity or systematic inbreeding [32]. As haplodiploid insects, bees have a single complementary sex determination (csd) locus that, when homozygous, causes production of sterile diploid males instead of females [33]. Long-term diploid male production further reduces effective population size and increases population genetic load [34] that can lead populations into an “extinction vortex” [35]. Therefore, selection for the evolution of mechanisms of inbreeding avoidance is expected to be strongly favored in bees, and other haplodiploid organisms. In diploid organisms, mechanisms of inbreeding avoidance include natal dispersal [36,37], or temporary dispersal to mate with individuals from other reproductive groups before returning to their natal sites to give birth [38,39].

One possible mechanism of inbreeding avoidance in bee species is pre-mating kin recognition through identification of cuticular hydrocarbons, or pheromones [40,41]. Bees exhibit intense male competition for female resources and males are the more discriminating sex [42]. For many species that nest gregariously, females are highly philopatric and the location and recognition of mates is based on male attraction by pheromones released from the female body. In this mating system, males actively prefer female odors from different populations to those from their own [43]. Therefore, male-mediated dispersal has been suggested as another possible mechanism to avoid the negative effects of inbreeding in solitary ground nesting bees that exhibit this suite of behavioral characteristics [40].

Another contributor to greater inbreeding in wild populations is reductions in population size due to habitat loss [44]. Urban areas often are characterized by high densities of humans and buildings, intensified land use, and extreme habitat loss. Therefore, plant and animal populations may exhibit higher levels of inbreeding in urban areas where landscape modifications can influence population connectivity by altering behavior, dispersal, and ultimately, distribution of genetic diversity and evolutionary potential of populations [45,46]. Indeed, investigating how landscape features shape evolution in human dominated habitats can be key to understanding adaptation and species responses to anthropogenic change [47]. Furthermore, understanding how populations are connected through gene flow in highly modified landscapes can inform management decisions and greatly improve efforts to enhance habitat connectivity [48].

**Fig 1:**
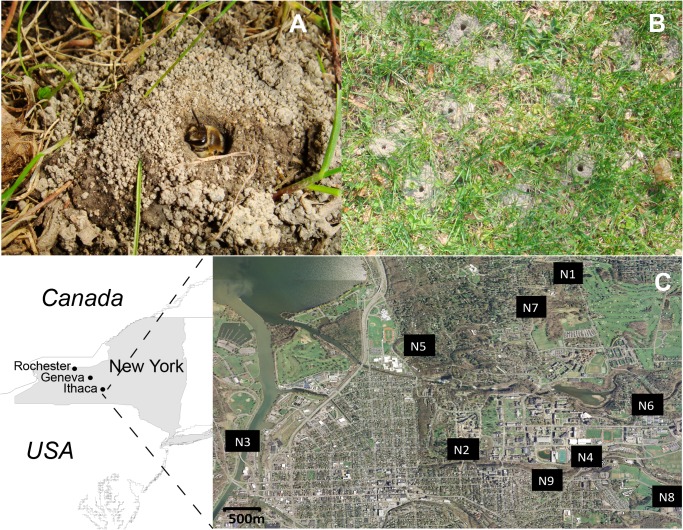
Nest aggregations of the ground nesting bee *Colletes inaequalis* selected for this landscape genetic study. (A) Solitary female at the entrance of her nest. (B) Nests of many individuals are often densely clustered within nesting sites. (C) Geographic location of sampled nest aggregations (N1-N9) throughout Ithaca (expanded map), and additional nest aggregations in Geneva and Rochester, New York, USA.

Due to the combined effects of urbanization and its unique behavioral ecology, one species likely to exhibit systematic effects of inbreeding is the ground-nesting bee *Colletes inaequalis*. This bee species is a wild pollinator of early blooming trees such as red maple and willow trees, and it is potentially important for blueberry and apple pollination in eastern North America [49]. *C*. *inaequalis* is solitary, meaning each female independently builds a nest (Fig 1A), and forages for nectar and pollen for provisioning of her offspring. Despite their solitary nature, females often nest in dense aggregations of up to 100 nests/m^2^ in open sandy soil (Fig 1B). *C*. *inaequalis* males and females are active for about 4 and 6 weeks of the year, respectively. Each spring, males emerge a few days before females and patrol nesting sites where males pounce on females to try to copulate when females first emerge from the ground, or later while entering and leaving their nest. Most mating is observed at the beginning of the season, prior to nest construction and pollen foraging [50]. Females construct their nests in the soil close to the points where they emerged just days earlier. Thus, the strong tendency for female natal philopatry and the patchy distribution of nesting habitats, especially in fragmented landscapes, could lead to persistent genetic isolation, differentiation among sites spaced close together, and ultimately inbreeding depression.

In this study, we investigate the fine scale genetic structure (<4 km) of *C*. *inaequalis* in an urban/suburban habitat in central New York, USA. Our approach combines information from highly polymorphic genetic markers and spatially explicit distribution models to test whether (1) individuals within a nest aggregation are inbred and more genetically related to each other than would be expected at random, (2) females within a nest aggregation are more genetically related than males, and (3) distribution and patchiness of suitable nesting habitat influence the distribution of genetic variability among nest aggregations in an urban landscape. Due to the ecological characteristics of this species, that include strong female philopatric behavior, short life spans and highly patchy distribution of nesting resources, we have three predictions about the fine-scale genetic structure of this species: (1) relatedness is higher within rather than among nest aggregations, (2) dispersal is male mediated, and (3) distance among nest aggregations is an important explanatory variable of genetic connectivity. Results from this study are relevant for bee conservation, especially as they provide insights into the mechanisms of inbreeding avoidance in solitary bees and how these play out in a human-modified landscape. Our findings also reveal landscape features necessary to maintain, and potentially enhance, bee population connectivity, minimize the risk of inbreeding depression in urban/suburban populations, and preserve the vital ecosystem services these bees provide. Finally, the management recommendations based on results from this study also have implications for many native species that share similar phenology and nest habitat specialization.

## Materials and Methods

### Ethics statement

Research sites sampled in this study included Cornell University property (N4, N6), private (N7, N9, N10) and public land (all other sites). Permissions to sample sites located on Cornell University and privately owned lands were respectively granted by the Cornell University grounds department and landowners. No permit was required for sites located on public land. Bees were not sampled from national parks or protected areas. *C*. *inaequalis* is not an endangered or protected species. Private land owners: Daniel Peck (N10), James Liebherr and Ann Hajek (N7), Mary Smith (N9). No permission was required for N1, N2, N3, N4, N5, N6, N8, and N11. Nest names (N1-N11) and their GPS coordinates are detailed on Table 1 of the manuscript.

**Table 1:** Geographic location and genetic diversity indices for the 11 sampled nest aggregations of *Colletes inaequalis* in NY, USA.

*Nest aggregation*	*Code*	*Coordinates*	*N*	*N*_*a*_	*A*_*r*_	*H*_*O*_	*H*_*S*_	*F*_*IS*_	*P-value*
Pleasant Grove	N1	42.4605N -76.4776	57	8.00	6.575	0.621	0.617	-0.006	0.5742
Ithaca Cemetery	N2	42.4444–76.4906	55	7.73	6.312	0.595	0.616	0.034	0.0788
Cass Park	N3	42.4458–76.5148	50	7.87	6.508	0.596	0.602	0.01	0.3418
Football Field	N4	42.4439–76.4768	53	7.60	6.228	0.597	0.62	0.037	0.0288
Cayuga Heights	N5	42.4568–76.4953	58	7.80	6.438	0.567	0.606	0.065	0.0015
Tunnel	N6	42.4495–76.4701	63	7.67	6.081	0.605	0.606	0.001	0.3967
Jim’s House	N7	42.4614–76.4831	52	7.67	6.317	0.603	0.607	0.007	0.3833
East Hill	N8	42.4390–76.4678	49	7.20	6.153	0.624	0.618	-0.01	0.6491
College Town	N9	42.4411–76.4737	48	8.07	6.670	0.607	0.625	0.028	0.117
Geneva	N10	42.7676–76.9856	24	6.87	6.597	0.616	0.625	0.014	0.347
Rochester	N11	42.1396–77.5687	39	6.93	6.314	0.215	0.624	0.655	0.0003

Symbols represent: number of individuals sampled (*N*), mean number of alleles (*N*_*a*_), allele richness (*A*_*r*_), observed heterozygosity (*H*_*O*_), heterozygosity within subpopulation (*H*_*S*_), inbreeding coefficient (*F*_*IS*_), and significance for *F*_*IS*_ adjusted for Bonferroni correction (5% level is 0.0003).

### Study area

We studied nest aggregations of *C*. *inaequalis* in urban/suburban areas of Ithaca, Geneva, and Rochester (New York, USA) (Fig 1A). Ithaca is a small city (14 km^2^, *ca*. 30,000 people) surrounded by rural areas consisting of agricultural fields, extensive forests, and scattered residences. The urban area and perimeter contain a large number of gardens and forested areas [51] that provide a wide variety of floral resources to bees during the spring and summer. Geneva is also a small urban area (11 km^2^, *ca*. 13,000 people) but, unlike Ithaca, it is surrounded by agricultural land dedicated mainly to apple, grape and pumpkin production. Rochester is a heavily urbanized area (93 km^2^, *ca*. 210,000 people) with a dense city park system within the perimeter of the city. The climate in Central New York is characterized by large variations in temperature throughout the year with cold winters reaching temperatures of -18°C, and warm and humid summers that reach 32°C.

### Spatial model

We informed our sampling scheme in the city of Ithaca by building a spatial suitability model to predict the distribution of nesting sites in the area. We chose biologically meaningful variables for our spatial model based on literature of nesting biology [50,52] of the target species and two known nesting sites (B. N. Danforth, pers. obs.). We considered that *C*. *inaequalis* requires at least three basic components to nest in a particular area: sparsely grassy areas, sandy soils [52], and south-facing slopes [50]. Thus, we generated binary layers (suitable *vs* unsuitable) for four landscape components: grassy areas, slope, aspect of the slope and soil type. To identify areas of the landscape with sparse grass, we performed an unsupervised classification of high resolution orthoimagery of the Ithaca area from 2007 (NYS Statewide Digital Orthoimagery Program Status), with a spatial resolution of. 3 m and. 6 m in urban and rural areas, respectively. We used ERDAS IMAGINE v.9 to classify the landscape into 20 categories based on intervals of wavelength in the grayscale. We chose four categories, as indicators of suitable habitat, based on the categories where two nest aggregations were located prior to model development. In addition, USGS digital elevation models, with a 10 m resolution, were used to classify areas with slope greater than 5° and aspect between 135° and 215°, as suitable nesting habitat for bees. To identify areas of suitable soil type, we used the Soil Survey Geographic Database (SSURGO) to select areas with >30% proportion of sandy soil. However, the soil type for the city of Ithaca is classified as suburban soil, with no further information about percent sand in the soil. Therefore, this fourth landscape variable was not included in our final model. All binary layers were overlaid in a simple multiplicative fashion in ArcGIS v.10.2, whereby the lack of suitability in any single spatial layer rendered a location unsuitable for nesting. The final map output was a suitability model that indicated suitable areas for nesting for *C*. *inaequalis* throughout an area approximately 40 km^2^ surrounding Ithaca, NY.

Due to the lack of fine-scale data for soil type inside the city, we analyzed soil samples in 11 nest aggregations in the city of Ithaca to gauge the soil type specificity of *C*. *inaequalis*. At each nest aggregation, we collected and mixed soil from three 30 cm core samples, after removing the top 5 cm from the sample to avoid confounding factors from topsoil additions. Subsequently, a soil texture analysis was performed to determine composition and percent sand in all the nesting sites.

### Model validation

We used two sampling approaches to estimate sensitivity (true positives) and specificity (true negatives) of the suitability model and to inform our field search for *C*. *inaequalis* nesting sites. First, we searched for 1) presence of a nest aggregation in areas predicted by the model, and 2) absence of nest aggregations in areas not predicted by the model. Twenty random points of each category were selected within our study area using Hawth’s tools [53]; all 40 of the randomly selected sites were checked for the presence of nest aggregations. In the second sampling approach, we selected from the model ten different zones throughout the study where high densities of potentially suitable nesting habitat occurred alongside roads. During the peak activity season, we drove slowly along these ten roads searching for any sign of bees or nest construction. In addition, because cemeteries are anecdotally known as preferred nesting sites for this species [52], we visited 10 cemeteries where the model indicated there was suitable habitat for nest aggregations.

To further validate our spatial model, we performed a bootstrap analysis to test whether the use of our spatial model increased the probability of finding suitable nesting sites. First we estimated the total area of suitable nesting habitat, as predicted by our model, in the vicinity of all known nesting areas, using 100 m and 300 m buffers. Then, we randomly chose 10,000 sites of equal area through a resampling procedure performed in Python 2.6, and compared these to the areas around the known nesting sites using a Kolmogorov-Smirnov (K-S) test performed in R. We ran the K-S test 10,000 times and calculated the proportion of statistically significant values indicating whether the amount of predicted suitable area was greater around known nesting sites than at randomly chosen sites. A higher proportion of significant values would support our choice of environmental variables that determine the distribution of *C*. *inaequalis* across the landscape and indicate a greater likelihood of finding nests when using our predictive model.

### Field sampling

For the genetic analysis, we collected a total of 548 female individuals of *C*. *inaequalis* using insect nets. Specimens were placed in vials with 95% ethanol and stored at -20°C until laboratory analysis. Sampled nest aggregations included sites within the suburban/urban areas of Ithaca (n = 9), Geneva (n = 1) and Rochester (n = 1) (New York, USA) (Fig 1C). We used Ithaca populations to examine the fine-scale genetic structure between nest aggregations, whereas inclusion of Geneva and Rochester allowed for the opportunity to investigate genetic structures at a regional scale. An average of 50 females (±10.7) were collected from each nest aggregation (Table 1) in April 2011 to ensure that all individuals belonged to the same generation. Linear distances between sampled nest aggregations in Ithaca ranged from 470 m to 4 km. Nest aggregations from Geneva and Rochester were located at 45 and 90 km away from Ithaca, respectively.

### Microsatellite genotyping

DNA was extracted from individual antennae using 150 μl of a 10% Chelex 100 solution, 5 μl of Proteinase K and incubated at 55°C for 1 h and at 99°C for 30 min [54]. Supernatant was used for the amplification of 18 microsatellite loci previously developed for *C*. *inaequalis* (CI010, CI12, CI15, CI23, CI27, CI028, CI35, CI62, CI66, CI73, CI075, CI87, CI98, CI099, CI102, CI106, CI131, CI179) that were multiplexed according to López-Uribe et al. [55]. PCR products were diluted and mixed with Hi-Di Formamide and GeneScan-500 LIZ for genotyping on an Applied Biosystems solution 3730xl DNA Analyzer (Applied Biosystems).

We estimated the power of the microsatellite markers to infer family relatedness using the software KinInfor v.1 [56]. The power for relationship inference (PWR) was estimated by the simulation procedure that is based on estimated allele frequencies from the microsatellite markers. Our primary objectives were to detect fullsibs (Δ_1_ = 0.5, Δ_2_ = 0.5), paternal halfsibs (Δ_1_ = 1, Δ_2_ = 0), maternal halfsibs (Δ_1_ = 0.5, Δ_2_ = 0) and unrelated (Δ_1_ = 0, Δ_2_ = 0) individuals. We ran 10^6^ simulated pairs of genotypes and set the confidence level at 0.05. We regenotyped 18% of our samples to estimate genotyping error, which varied from 0 to 0.02 depending on the locus (S2 Table). To identify the estimator for which our dataset contains the most information and the greatest power of relatedness detection, we used the reciprocal of the mean squared deviations (RMSD) of different relatedness estimators Ritland et al. [57], Li et al. [58], Queller and Goodnight [59], Lynch and Ritland [60], and Wang [61].

### Genetic diversity and relatedness

Presence of null alleles and large allele dropouts were detected in MicroChecker v.2.2.3 [62]. Hardy-Weinberg and linkage disequilibrium were tested for using the exact test incorporated in GENEPOP [63]. We characterized the genetic diversity of each nest aggregation after removing non-neutral loci by calculating the mean number of alleles (*N*_*a*_), observed and expected heterozygosity (*H*_*O*_ and *H*_*S*_), using the software GenoDive v.2.0b25 [64]. Allele richness, corrected for sample size, and inbreeding coefficients (*F*_*IS*_) were estimated in FSTAT v.2.9.3.2 [65]. Sibship among females within nest aggregations was estimated using the software COLONY v.2.0.4.4 [66] by running two chains using the very high precision method, assuming a polygamous mating system and presence of inbreeding. We removed full sibs from our dataset for all the downstream analyses to have unbiased estimators of population structure from unrelated individuals.

### Tests for deviations from random mating

We used three different methods to test the hypothesis of non-random mating between individuals within and between nest aggregations. First, we tested for the overall population genetic structure using three G-statistics (Nei’s *G*_*ST*_, Hedrick’s *G*_*ST*_, Jost’s *D*_*est*_) in GenoDive v.2.0b25 [64]. Pairwise Nei’s *G*_*ST*_ was estimated using the *fstat* function in the R package “adegenet” [67], which estimates genetic differentiation based on information from allele frequencies and heterozygosity. Significance was assessed by a permutation test with 10000 repetitions. In the second approach, we estimated pairwise relatedness between individuals, to test whether mean relatedness within nest aggregations was higher than among nest aggregations. We used Queller and Goodnight’s relatedness estimators (*QGt*) because of its robustness to differences in the number of alleles per locus, the allele frequency distribution, sample size, and the presence of inbreeding [61]. Furthermore, the KinInfor analysis identified this estimator as the one for which our dataset contains the greatest power of relatedness detection (see results). Significant differences in average relatedness within and between nest aggregations were assessed through the bootstrapping method implemented in COANCESTRY v.1.0.1.1 [68]. Last, we investigated the spatial autocorrelation between individuals every 0.5 km, using the software SPAGeDi [69]. Standard errors were estimated by jackknifing over loci. Confidence intervals around the null expectation of no genetic differentiation were assessed by permuting 1000 multi-locus genotypes and spatial coordinates.

### Effective population size and dispersal

Contemporary effective population sizes were calculated using the sibship assignment method implemented COLONY v.2.0.4.4 [66]. This method estimates effective population size based on the frequency of sibs within a subpopulation with respect to the whole population, while correcting for deviations from non-random mating [70]. We also used the software NeEstimator v.2 [71] to estimate effective population sizes using other three methods: Coancestry method [72], heterozygote-excess method [73] and linkage disequilibrium method [74].

To test the hypothesis of male-mediated dispersal, we reconstructed parental genotypes from female offspring genotypes using the full maximum likelihood method in the software COLONY v.2.0.4.4 [66]. The algorithm used in COLONY generates posterior probabilities for the best five hypothesized parental genotypes for each individual. We used these parental genotypes to infer relatedness among females and among males in the previous generation in each nest aggregation. From all parental genotypes, we only included in our analyses genotypes with a posterior probability >0.75 and removed individuals with less than five accurately inferred genotypes [75]. Therefore, for downstream analyses, we were limited to the number of parental genotypes that we could confidently assign based on the above criteria. Relatedness between female and male individuals in each nest aggregation was quantified using the *QGt’s* point estimator [59]. Significant differences in relatedness between females and males across nest aggregations were determined using a one-way ANOVA, assuming unequal variance among samples.

### Landscape genetics analysis

We calculated the area of each nest aggregation as the minimal area that encompassed all nest entrances at each site, and statistically tested this variable as a predictor of within nest aggregation genetic diversity (effective population size, number of alleles and expected heterozygosity). Pairwise Euclidean distance between nests was tested as a predictive variable of genetic differentiation (pairwise G_ST_) using Mantel tests, as implemented in program zt [76]. To build explicit landscape genetic models, we merged the spatial and genetic information of each nest aggregation. We used the inverse distance weighted (IDW) values among nest aggregations as an interpolation method to visualize the landscape map of (1) Euclidean distance (geographic distance only), (2) inbreeding coefficient (*F*_*IS*_) and (3) genetic composition (spatial principal components, sPCA) of *C*. *inaequalis* within the Ithaca study area. The sPCA analysis, performed in the R package “adegenet” [67], combines information from genotypes and spatial distribution of individuals to identify distinct genetic clusters within a group of individuals. All geospatial operations at the landscape scale were performed in ArcGIS v.10.2, and all statistical analyses were performed in R.

## Results

### Spatial model

**Fig 2:**
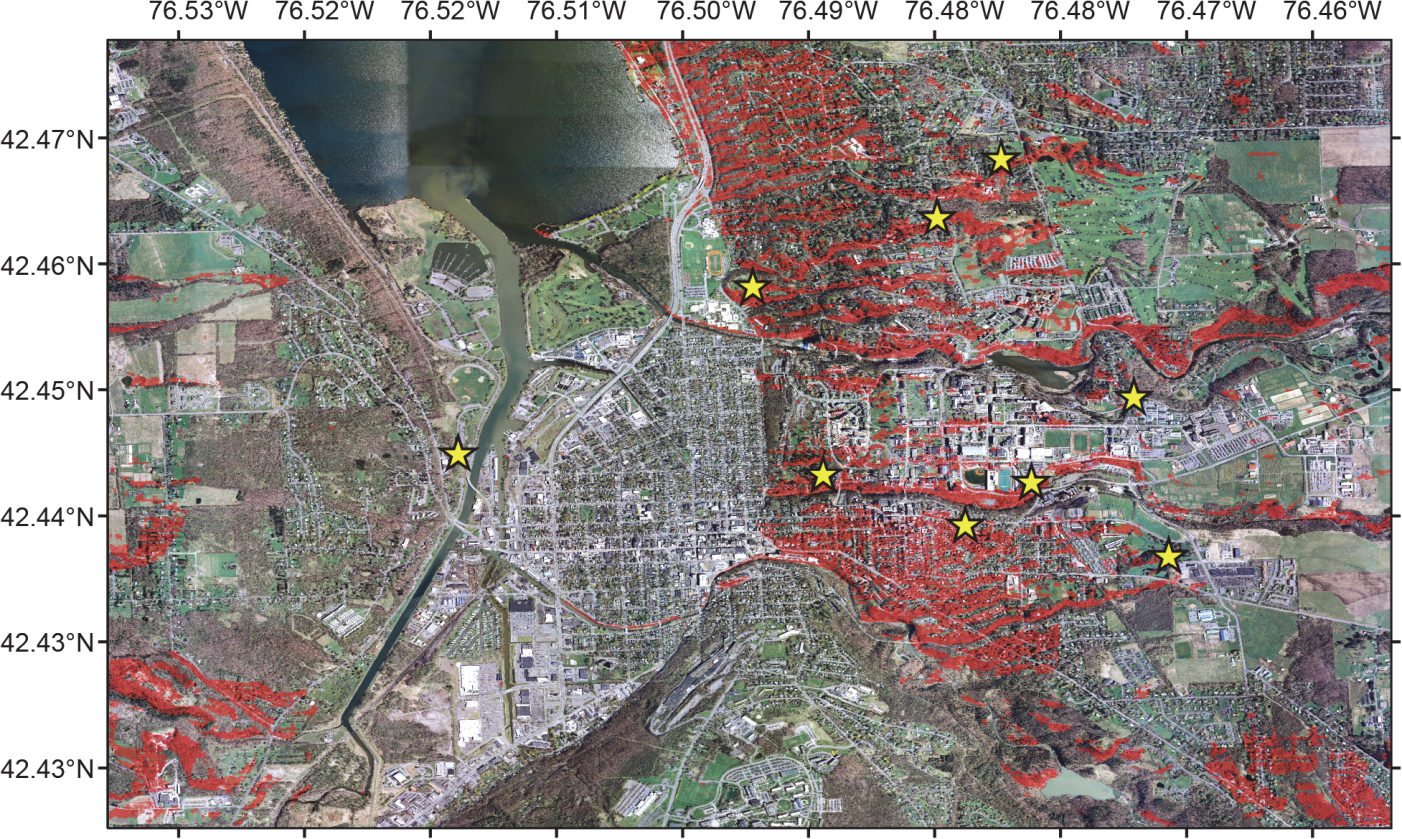
Predictive spatial model indicating suitable nesting habitat for *Colletes inaequalis* in Ithaca study area (NY, USA). Red indicates suitable nesting habitat predicted by the model. Yellow stars indicate presence of nest aggregations.

Our predictive spatial model indicated that 24% of the area in the city of Ithaca could be suitable nesting habitat for *C*. *inaequalis* (Fig 2). Using the spatial model that we built based on the landscape characteristics of two known nest aggregations, we identified 13 new nest aggregations using surveys along roads and cemeteries as the main sampling scheme. For the genetic analysis, we used 9 of the 15 nest sites, due to the close geographic proximity between some sites. Fourteen out of the 15 nesting sites occurred exactly in areas predicted to be suitable nesting habitat by our spatial model, which indicates a high model sensitivity. We also searched for nests at 40 randomly selected locations deemed to be suitable and found no nests exactly at those points, indicating a low model specificity. The lack of available landscape-level soil data may have contributed to the low specificity, given the apparent highly selective nature of *C*. *inaequalis* for sandy soils in the areas where they nest. Results of our soil texture analysis from the nest sites revealed that sand comprised a mean of 61% and never less than 30% (SD = 14.5) of the particles in the soil samples (S1 Table). Our model was informative about the spatial location of suitable nesting habitat for *C*. *inaequalis*. From the bootstrap analysis, the K-S test revealed that the amount of predicted suitable area in the neighborhood of observed nest sites was greater than around random sites, and this was true within distances of 100 and 300 m (*P*_100_< 0.0001; *P*_300_< 0.0001).

### Genetic diversity

Significant evidence of null alleles was detected for loci CI35 and CI87 across all nest aggregations (S1 Table); therefore, these loci were excluded from all analyses. All loci from the nest aggregation in Rochester exhibited Hardy-Weinberg disequilibrium (deficit of heterozygotes). Excluding this site, nine loci (CI27, CI12, CI66, CI62, CI099, CI075, CI15, CI23, CI179) exhibited no significant deviations from Hardy-Weinberg across all nest aggregations, and seven loci (CI028, CI131, CI73, CI106, CI102, CI98) exhibited significant heterozygote deficiency in no more than three nest aggregations (S2 Table). The remaining 16 loci were at linkage equilibrium, except for locus CI102, which was excluded from all analyses. Queller and Goodnight’s (65) was identified as the most informative relatedness estimator with an estimated power for relationship inference (PWR) of 0.838, based on 15 neutral loci in our dataset.

The microsatellite loci showed an average number of alleles (*Na*) of 7.73 (±0.443), allele richness (*A*_*r*_) corrected for sample size of 6.38 (±0.191), and observed and expected heterozygosity of 0.568 (±0.055) and 0.615 (±0.061), respectively (Table 1). After Bonferroni correction, significant heterozygote deficiencies were only found in the nest aggregation from Rochester (Table 1). The COLONY analysis detected five full sibs in our dataset (nest aggregations N1, N5, N6, N9 and N10). One individual of these dyads was randomly removed for all downstream analyses.

### Non-random mating

All G-statistic estimators indicated significant (p<0.005) but weak genetic structure (Nei’s *G*_*ST*_ = 0.011; Hedrick’s *G*_*ST*_ = 0.032; Jost’s *D*_*est*_ = 0.019), suggesting deviations from random mating across the study area. For pairwise Nei’s *G*_*ST*_, 10 out of the 36 comparisons were significantly different than zero (S3 Table). When nest aggregations from Geneva and Rochester were included, higher but still weak genetic differentiation was detected (Nei’s *G*_*ST*_ = 0.017; Hedrick’s *G*_*ST*_ = 0.047; Jost’s *D*_*est*_ = 0.029).

**Fig 3:**
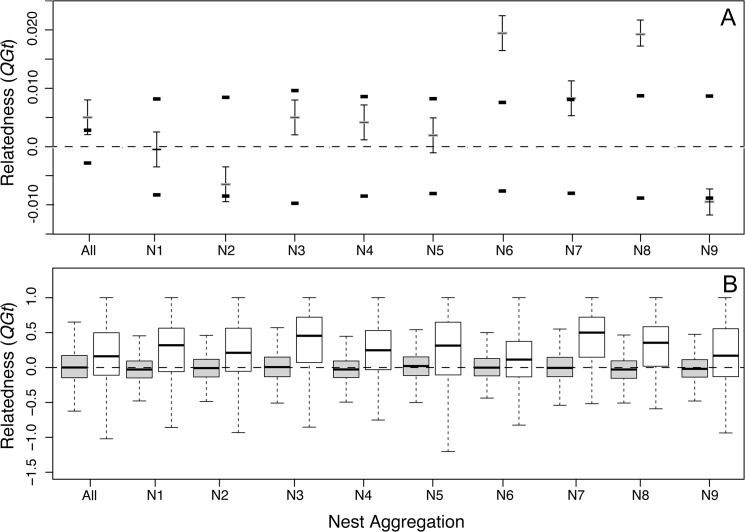
Relatedness (Queller and Goodnight’s, *QGt*) among individuals within nest aggregations. (A) Mean relatedness among the total number of individuals (All) and individuals within each nest aggregation (N1—N9). Bars represent standard error, and bounding short lines represent the 95% confidence interval expected under panmixia. (B) Boxplots comparing relatedness between males (grey) and females (white) within nest aggregations across the Ithaca landscape. Differences between males and females were highly significant (*F* = 1244.16, *P*< 0.001); females were more genetically related than males at all nest sites.

**Fig 4:**
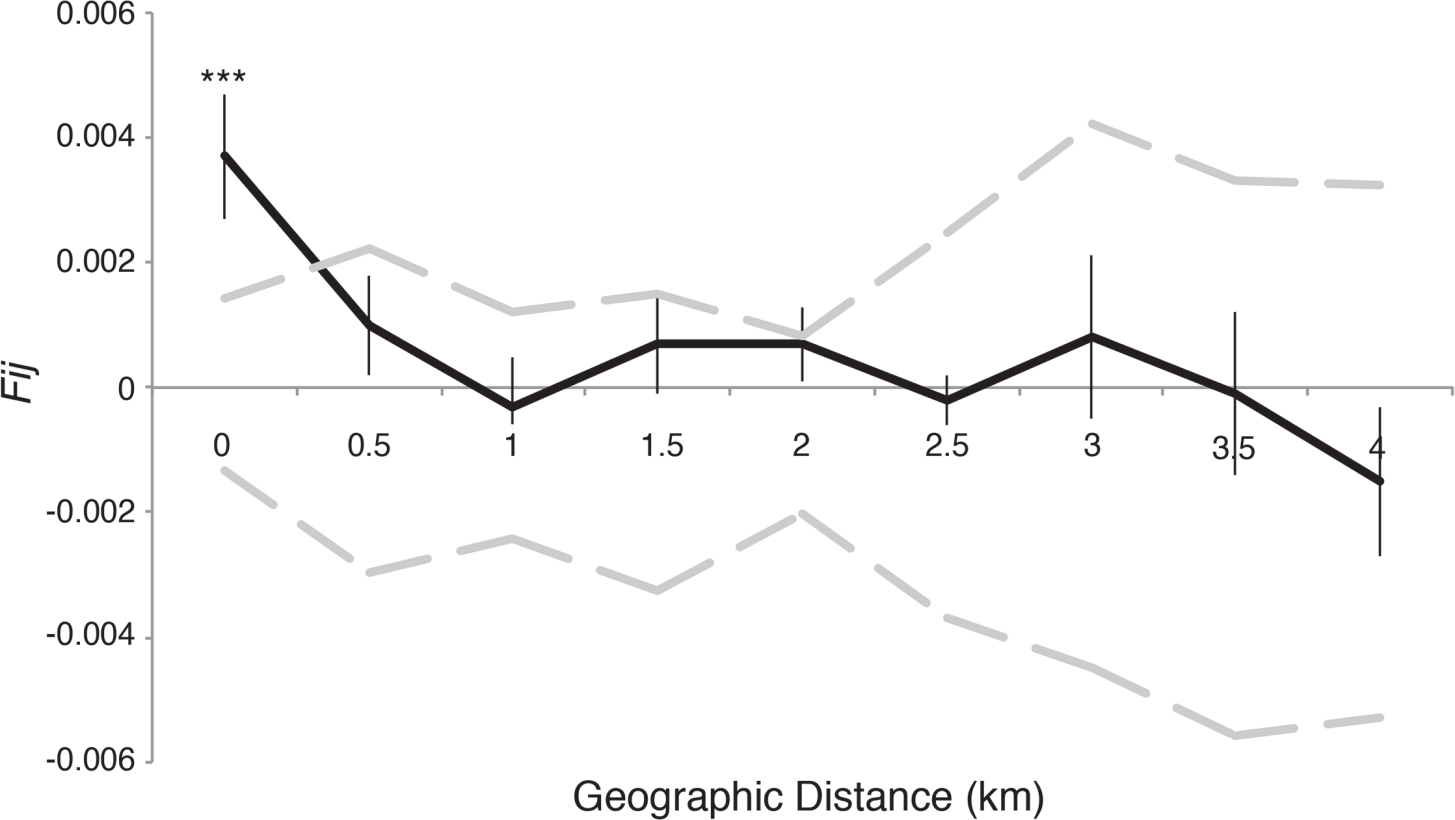
Spatial autocorrelation of kinship *vs* geographic distance. The black line represents the mean of all pairwise kinship coefficients between individuals at different distance intervals. Gray lines depict the 95% confidence intervals showing the expected range of *F*_*IJ*_ if there was no correlation between kinship and distance. Individuals at distances lower than 0.5 km show higher genetic relatedness than would be expected by random mating.

Mean levels of relatedness within nest aggregations were low and exhibited large variances (*QGt* = 0.003± 0.0134). Overall, average relatedness was higher among individuals within nest aggregations than among randomly chosen individuals *(P<* 0.01). However, the difference in relatedness between groups was variable when each nest was assessed independently (Fig 3A). The spatial autocorrelation analysis further supported evidence of non-random mating among individuals from different nest aggregations. The autocorrelogram shows significant positive genetic correlations among individuals at a spatial scale smaller than 0.5 km (Fig 4).

### Effective population size and dispersal

Effective population sizes based on the sibship assignment method ranged from 55 to 71 across all nest aggregations (*mean* = 60). Other methods of estimation resulted in ‘infinite’ population sizes, suggesting that larger sample sizes would be necessary for accurate parameter estimation [71]. We were able to reconstruct with confidence the parental genotypes of 221 females and 490 males, and found a significantly higher relatedness within each nest aggregation among females than among males *(P<* 0.0001) (Fig 3B).

### Landscape analysis

**Fig 5:**
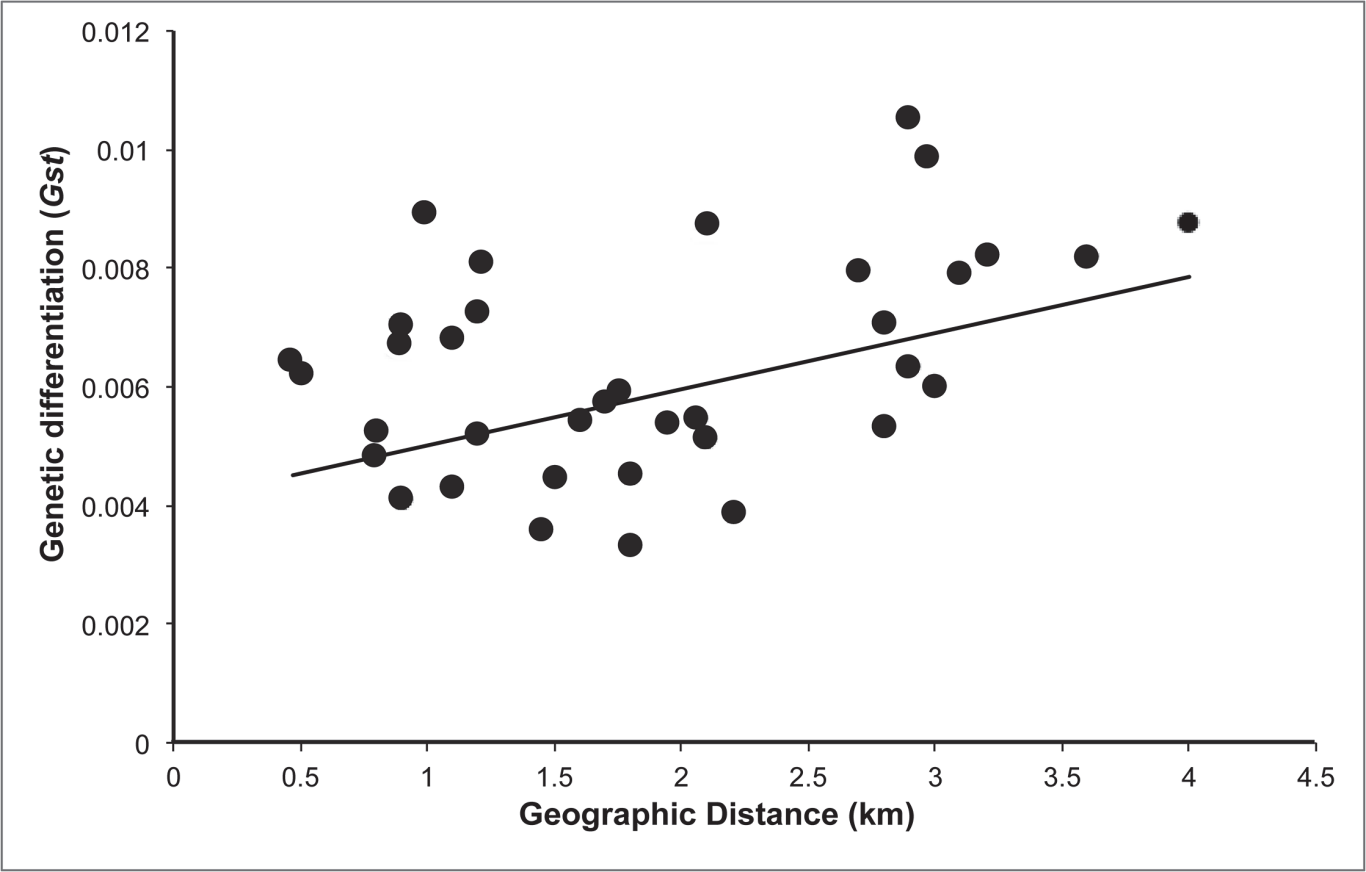
Plot of isolation by distance. Relationship between pairwise genetic differentiation (*G*_*ST*_) and linear geographic distance (km) for the nine nest aggregations in Ithaca, NY. Mantel test was not significant for the 9 nest aggregations (*r* = 0.37, *P* = 0.096).

**Fig 6:**
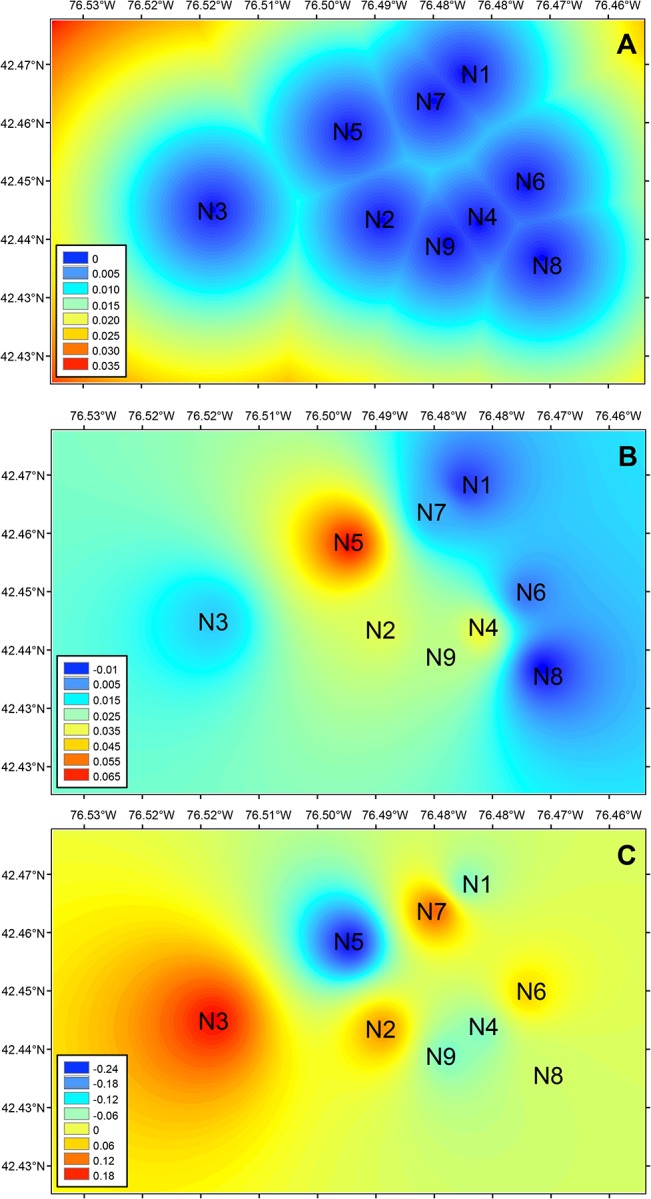
Spatial representation of interpolated landscape genetics metrics for *Colletes inaequalis* across Ithaca (NY, USA). Inverse distance weighting of (A) Euclidean distance alone, (B) inbreeding coefficient (*F*_*IS*_), and (C) spatial principal components (sPCA) between nest aggregations. Similar colors represent the similarity of individuals in the landscape based on the three different parameters measured. The genetic landscape of *C*. *inaequalis* in the Ithaca study area is not only explained by geographic distance among nests. Nest aggregation N3 is the most geographically isolated, but N5 exhibits the highest inbreeding coefficient (B) and, correspondingly, is the most genetically different (C).

We found a significant correlation between size of nesting patch and effective population size (*r* = 0.71, *P* = 0.03), but not with number of alleles or expected heterozygosity. The relationship between F_ST_ and geographic distance showed a positive trend but non-significant trend, partially explaining the genetic differentiation between nest aggregations across the landscape (*r* = 0.37, *P* = 0.096) (Fig 5). The plotted landscape genetic attributes of the nest aggregations showed that based on just Euclidean distance, the population that is most geographically isolated from the others is N3 (Fig 6A). However, N3 does not appear to be the nest aggregation with highest inbreeding coefficient (Fig 6B). Rather, the overall pattern of inbreeding across the landscape is that when moving from a rural to a more urbanized setting, inbreeding tends to increase. This pattern is visually evident from the distinct band of higher inbreeding in nest aggregations located in areas of Ithaca with higher urbanization. Accordingly, in Rochester, the most urbanized site, the sampled nest aggregation exhibited extremely high levels of inbreeding, with a value greater than tenfold of those from Ithaca (Table 1). Results from the landscape map of sPCA revealed that N5, the nest aggregation with highest inbreeding in Ithaca, is correspondingly the most genetically different, with the rest of the nest aggregations exhibiting more similar genetic compositions (Fig 6C).

## Discussion

Our results from G-statistics, relatedness, and spatial autocorrelation support the hypothesis that *C*. *inaequalis* mate with individuals from the same nest aggregation more frequently than expected under panmixia. In fact, we detected significant genetic differentiation among nest aggregations sampled within a ~14 km^2^ area, demonstrating that solitary bee populations can show genetic structure at small geographic scales despite their ability to fly long-range [77]. These results differ from other studies of solitary and primitively eusocial bees with similar body sizes, which have found weak or no genetic structure at broader geographic scales [17,78–80]. However the deviations from random mating were, for the most part, mild. In some nest aggregations levels of relatedness were lower than expected. A deeper understanding of these patterns at fine geographic scales is needed to make informed decisions for keeping the necessary environmental features that maintain long term population stability of solitary bees across human-altered landscapes. Because population genetic patterns are dynamic, our inferences about the degree of random mating are limited to the generation that we sampled. Nevertheless, the observed lack of strong population structure suggests significant genetic exchange between nest aggregations over time.

One factor that could reduce the long term stability of bee populations across the landscape would be high levels of inbreeding. Indeed, presence of intranidal mating in several solitary bees with gregarious nesting [21,81] suggests that inbreeding may be a widespread phenomenon among bees, due to their high fidelity to natal sites and patchy distribution of resources [82]. The detrimental effects of inbreeding would likely be exacerbated by potential production of high frequencies of diploid males that can increase genetic load in populations. In this study, we found that, except for one nest aggregation from a highly urbanized area in Rochester, NY, we did not detect significantly high levels of inbreeding. The observed absence of inbreeding despite non-random mating at a fine geographic scale may be the result of one or a combination of the following mechanisms: (1) maintenance of large effective population sizes in nest aggregations that allow for low probability of mating between sibs; (2) negative assortative mating based on kin recognition that decreases mating between relatives; and (3) high levels of gene flow among nest aggregations that reduce levels of coancestry within each nest aggregation. Any combination of these factors could counteract the potential for increased levels of inbreeding.

The first mechanism, maintenance of large effective population sizes, is not strongly supported by our data. On average, effective population sizes of *C*. *inaequalis* nest aggregations were small (~60 individuals) and significantly lower than census population sizes. In most sampled nest aggregations, we observed number of individuals (census population sizes) that varied between hundreds to thousands per site (M. M. López-Uribe pers. obs.). Presence of negative assortative mating, the second possible mechanism of inbreeding avoidance, has been demonstrated in the congeneric European species *C*. *cunicularius* [43]. However, the ability to discriminate female sex pheromones appears to be limited in *C*. *inaequalis* as males of this species are attracted to females of the closely related species *C*. *validus* [50]. Therefore, maintenance of large effective population sizes or negative assortative mating within the nesting aggregations may not be the main factors explaining lack of high levels of inbreeding in this species.

We hypothesize that the most likely demographic process that explains the observed pattern of low genetic structure and lack of inbreeding is male-based dispersal. Within nesting sites, females were more genetically related than males, indicating a sex bias in migration rates. Sex-biased dispersal is a common behavior in mammals, birds and reptiles [36,82] and has recently been demonstrated in bees [83]. However, the ecological and evolutionary drivers of male-mediated gene flow in bees are not well understood. Bee mating systems are generally thought to be monandrous in females and polygamous in males, even though genetic data supporting this premise are lacking [84]. In monandrous systems, receptive females are considered a limited resource, thus male dispersal is expected as a strategy to maximize their reproductive success. However, *C*. *inaequalis* females are receptive for most of their lives and can probably mate more than once [50]. Because female philopatry in *C*. *inaequalis* may dramatically increase coancestry of individuals at natal sites and males have the capacity for kin discrimination [43], we interpret that male-biased dispersal in this bee species is favored to avoid kin competition [85] and long-term inbreeding, as has been predicted by theoretical models for species with this kind of mating system [86].

Three aspects of our results highlight the importance of maintaining high densities of suitable nesting patches distributed across the landscape for the enhancement of solitary bee populations in urban areas. First, we detected a positive trend between genetic structure and geographic distance among nest aggregations, suggesting that nest aggregations too far apart could become genetically isolated. Second, effective population sizes of the nest aggregations were positively correlated with the size of the nesting patches. Thus, it is likely that increasing the amount of available suitable nesting habitat in the landscape may increase bee population abundance and maintain long term population stability. Third, females of *C*. *inaequalis*, and possibly other ground nesting solitary bees, disperse less than males and may have limited colonization capacity, especially where suitable nesting habitat is limited and patchily distributed. Management strategies for bee conservation generally focus on improving habitat quality for females because they provide the majority of pollination services, while males are short-lived. Our results draw attention to the important role of males for bee population dynamics and long-term persistence, as they are likely the mediators of population connectivity.

Urbanization can negatively impact local and regional levels of gene flow in native bees [13]. Even though we did not explicitly sample nest aggregations across a gradient of urbanization, we found higher levels of inbreeding with increased urbanization, and the nest aggregation from the most heavily urbanized area (Rochester, NY) showed a tenfold increase in inbreeding. However, data from other nest aggregations located in heavily urbanized areas are necessary to corroborate any potential generality of this finding. Studies of urban ecology have demonstrated that species diversity can peak in transitional regions between urban and rural areas [87] and human activity can increase landscape heterogeneity to a certain degree. Still, high degrees of urbanization have a strong homogenizing effect that dramatically lowers species diversity [88]. In the case of bees, urban landscapes can actually provide suitable habitat for pollen generalists due to the wide variety of floral resources that these habitats provide throughout the year [89,90]. In addition, cavity-nesters show a positive effect to urbanization because of increased suitable nesting areas in human-made structures [91]. However, for ground nesting bees, suitable nesting sites are likely the limiting resource in urban landscapes where soil is heavily modified and the proportion of bare soil is dramatically reduced [92]. Given our results showing the critical connection between amount of suitable nesting habitats and native bee abundance, this study has direct implications for conservation and management. In order to enhance and maintain populations of these native bees in urban/suburban landscapes, it may be necessary to increase the distribution and density of sites with bare, undisturbed and sandy soil. In addition, due to the possible low colonization rates of solitary bees, seeding suitable nesting sites with bee larvae may be an effective approach to increase pollination services in human-modified landscapes.

## Supporting Information

S1 Table Particle composition analysis of soil samples from studied nesting sites of *Colletes inaequalis*. Particles are expressed in percent sand, clay and silt.
(DOCX)

S2 Table Descriptive summary and genetic diversity indices of the 18 microsatellite loci. Number of alleles (*N*_*a*_), observed heterozygosity (*H*_*O*_), heterozygosity within subpopulation (*H*_*T*_), inbreeding coefficient (*H*_*DEF*_), genotyping error and significance of null alleles. Stars denote levels of significance for the presence of null alleles (* *P*< 0.1; ** *P*< 0.05; *** *P*< 0.01).
(DOCX)

S3 Table Pairwise genetic differentiation among nest aggregations in Ithaca. Nei’s *Gst* values based on 15 microsatellite markers (below) and pairwise distance among nest aggregations (above). Numbers in bold denote significant differentiation at the alpha-level 0.05.
(DOCX)
