# Supplementary material for: Nest Suitability, Fine-Scale Population Structure and Male-Mediated Dispersal of a Solitary Ground Nesting Bee in an Urban Landscape
Source: PLoS One. 2015 May 7;10(5):e0125719. doi: 10.1371/journal.pone.0125719 (PMC4423849; doi:10.1371/journal.pone.0125719)
Supplement: S1 Table — Particles are expressed in percent sand, clay and silt. (DOCX) [file pone.0125719.s001.docx]

**S1 Table. Particle composition analysis of soil samples from studied nesting sites of *Colletes inaequalis*.** Particles are expressed in percent sand, clay and silt.

| ***Site*** | ***Sand*** | ***Clay*** | ***Silt*** |
| --- | --- | --- | --- |
| Pleasant Grove | 71.5 | 6.9 | 21.6 |
| Ithaca Cemetery | 39.9 | 11.9 | 48.2 |
| Cass Park | 74.5 | 3.7 | 21.8 |
| Football Field | 34.8 | 18.3 | 46.9 |
| Cayuga Heights | 67.7 | 10 | 22.3 |
| Tunnel | 57 | 5.5 | 37.5 |
| Jim’s House | 76.3 | 5.4 | 18.3 |
| East Hill | 50.3 | 10.2 | 39.5 |
| Ctown | 64.2 | 9.8 | 25.9 |
| Uris Hall | 77.2 | 7.6 | 15.3 |
| A-lot | 60.8 | 8.9 | 30.3 |
