# Supplementary material for: Nest Suitability, Fine-Scale Population Structure and Male-Mediated Dispersal of a Solitary Ground Nesting Bee in an Urban Landscape
Source: PLoS One. 2015 May 7;10(5):e0125719. doi: 10.1371/journal.pone.0125719 (PMC4423849; doi:10.1371/journal.pone.0125719)
Supplement: S2 Table — Number of alleles (N a), observed heterozygosity (H O), heterozygosity within subpopulation (H T), inbreeding coefficient (H DEF), genotyping error and significance of null alleles. Stars denote levels of significance for the presence of null alleles (* P< 0.1; ** P< 0.05; *** P< 0.01). (DOCX) [file pone.0125719.s002.docx]

**Table S2. Descriptive summary and genetic diversity indices of the 18 microsatellite loci.** Name of locus, annealing temperature (T_a_), allele sizes, number of alleles (*Na*), observed heterozygosity (*Ho*), heterozygosity within subpopulation (*H_T_*), significance for heterozygote deficiency (*H_DEF_*), population where heterozygous deficiencies were found (Pop Het Def), estimated genotyping error and significant presence of null alleles. Stars denote levels of significance for the presence of null alleles (* *P*< 0.1; ** *P*< 0.05; *** *P*< 0.01).

| **Locus** | **T_a_** | **Allele sizes** | **N_a_** | **H_O_** | **H_T_** | ***H_DEF_*** | **Pop *H_DEF_*** | **Genotyping**  **Error*^a^*** | **Significance**  **Null Alleles** |
| --- | --- | --- | --- | --- | --- | --- | --- | --- | --- |
| CI028 | 59 | 160 - 194 | 5 | 0.748 | 0.908 | 0.2086 | N1, Rochester | 0.001 |  |
| CI27 | 55 | 207 - 239 | 11 | 0.650 | 0.791 | 0.0016 | Rochester | 0.001 |  |
| CI12 | 58 | 114 - 132 | 12 | 0.463 | 0.570 | 0.0002 | Rochester | 0.001 | * |
| CI131 | 59 | 248 - 270 | 3 | 0.694 | 0.853 | 0.0058 | N5, Rochester | 0.001 |  |
| CI66 | 54 | 193 - 223 | 16 | 0.326 | 0.366 | 0.0092 | Rochester | 0.02 |  |
| CI73 | 55 | 142 - 164 | 6 | 0.434 | 0.476 | 0.0008 | N5, Rochester | 0.001 | * |
| CI35 | 54 | 238 - 314 | 13 | 0.188 | 0.330 | 0 | All | 0.001 | *** |
| CI106 | 53 | 119 - 164 | 25 | 0.654 | 0.767 | 0 | N2, Geneva, Rochester | 0.001 |  |
| CI62 | 55 | 350 - 410 | 15 | 0.744 | 0.853 | 0.002 | Rochester | 0.001 |  |
| CI102 | 54 | 181 - 205 | 12 | 0.051 | 0.058 | 0 | N1, Rochester | 0.001 |  |
| CI099 | 54 | 367 - 395 | 6 | 0.279 | 0.331 | 0.0329 | Rochester | 0.001 |  |
| CI87 | 54 | 304 - 356 | 30 | 0.690 | 0.908 | 0 | All | 0.001 | *** |
| CI075 | 59 | 221 - 249 | 10 | 0.621 | 0.779 | 0 | Rochester | 0.001 |  |
| CI15 | 52 | 250 - 264 | 8 | 0.588 | 0.705 | 0.0001 | Rochester | 0.001 |  |
| CI010 | 53 | 169 - 197 | 18 | 0.669 | 0.813 | 0 | Rochester | 0.02 | * |
| CI98 | 55 | 350 - 410 | 19 | 0.640 | 0.803 | 0 | N1, N3, N4, Rochester | 0.001 | * |
| CI23 | 55 | 240 - 261 | 13 | 0.412 | 0.473 | 0 | Rochester | 0.001 |  |
| CI179 | 59 | 299 - 305 | 4 | 0.519 | 0.629 | 0.1046 | Rochester | 0.001 |  |

*^a^* A conservative genotyping error rate of 0.001 was used for loci for which we did not detect genotyping errors.
