# Supplementary material for: Nest Suitability, Fine-Scale Population Structure and Male-Mediated Dispersal of a Solitary Ground Nesting Bee in an Urban Landscape
Source: PLoS One. 2015 May 7;10(5):e0125719. doi: 10.1371/journal.pone.0125719 (PMC4423849; doi:10.1371/journal.pone.0125719)
Supplement: S3 Table — Nei’s Gst values based on 15 microsatellite markers (below) and pairwise distance among nest aggregations (above). Numbers in bold denote significant differentiation at the alpha-level 0.05. (DOCX) [file pone.0125719.s003.docx]

**S3 Table. Pairwise genetic differentiation among nest aggregations in Ithaca.** Nei’s Gst values based on 15 microsatellite markers (below) and pairwise distance among nest aggregations (above). Numbers in bold denote significant differentiation at the alpha-level 0.05.

|  | Plea_Grove (N1) | Itha_Ceme (N2) | Cass_Park (N3) | Foot_Field (N4) | Cayu_Heig (N5) | Tunnel (N6) | Jim_House (N7) | East_Hill (N8) | Ctown (N9) |
| --- | --- | --- | --- | --- | --- | --- | --- | --- | --- |
| (N1) | - | 0.9 | 2.8 | 1.8 | 1.5 | 1.1 | 0.5 | 3 | 2.8 |
| (N2) | 0.0041 | - | 2.06 | 1.2 | 1.45 | 1.8 | 0.8 | 1.94 | 0.89 |
| (N3) | 0.0071 | 0.0055 | - | 3.2 | 1.75 | 3.6 | 2.7 | 4 | 2.95 |
| (N4) | 0.0033 | 0.0052 | **0.0083** | - | 2.1 | 0.79 | 1.7 | 0.89 | 0.47 |
| (N5) | 0.0045 | 0.0036 | 0.0059 | 0.0051 | - | 2.2 | 1.6 | 2.9 | 2.1 |
| (N6) | 0.0043 | 0.0045 | **0.0082** | 0.0049 | 0.0039 | - | 1.1 | 1.2 | 1.2 |
| (N7) | 0.0062 | 0.0053 | **0.0080** | 0.0058 | 0.0054 | 0.0068 | - | 3.1 | 2.9 |
| (N8) | 0.0060 | 0.0054 | **0.0088** | 0.0071 | 0.0063 | **0.0072** | 0.0079 | - | 1 |
| (N9) | 0.0054 | 0.0068 | **0.0100** | 0.0066 | **0.0088** | **0.0081** | **0.0106** | **0.0090** |  |
